# Perception of visual apparent motion is modulated by a gap within concurrent auditory glides, even when it is illusory

**DOI:** 10.3389/fpsyg.2015.00564

**Published:** 2015-05-19

**Authors:** Qingcui Wang, Lu Guo, Ming Bao, Lihan Chen

**Affiliations:** ^1^Hangzhou Applied Acoustics Research Institute, Key Laboratory of Science and TechnologyHangzhou, China; ^2^Institute of Acoustics – Chinese Academy of SciencesBeijing, China; ^3^Department of Psychology and Key Laboratory of Machine Perception (Ministry of Education), Peking UniversityBeijing, China

**Keywords:** gap transfer illusion, Ternus display, intra-modal, cross-modal, perceptual grouping

## Abstract

Auditory and visual events often happen concurrently, and how they group together can have a strong effect on what is perceived. We investigated whether/how intra- or cross-modal temporal grouping influenced the perceptual decision of otherwise ambiguous visual apparent motion. To achieve this, we juxtaposed auditory gap transfer illusion with visual Ternus display. The Ternus display involves a multi-element stimulus that can induce either of two different percepts of apparent motion: ‘element motion’ (EM) or ‘group motion’ (GM). In “EM,” the endmost disk is seen as moving back and forth while the middle disk at the central position remains stationary; while in “GM,” both disks appear to move laterally as a whole. The gap transfer illusion refers to the illusory subjective transfer of a short gap (around 100 ms) from the long glide to the short continuous glide when the two glides intercede at the temporal middle point. In our experiments, observers were required to make a perceptual discrimination of Ternus motion in the presence of concurrent auditory glides (with or without a gap inside). Results showed that a gap within a short glide imposed a remarkable effect on separating visual events, and led to a dominant perception of GM as well. The auditory configuration with gap transfer illusion triggered the same auditory capture effect. Further investigations showed that visual interval which coincided with the gap interval (50–230 ms) in the long glide was perceived to be shorter than that within both the short glide and the ‘gap-transfer’ auditory configurations in the same physical intervals (gaps). The results indicated that auditory temporal perceptual grouping takes priority over the cross-modal interaction in determining the final readout of the visual perception, and the mechanism of selective attention on auditory events also plays a role.

## Introduction

In a noisy environment, we often need to integrate various sources of information, including spatial, temporal, and semantic cues between multiple signals to build a coherent representation for the target sensory events (Bertelson and Aschersleben, 2003; Alais and Burr, 2004; Stein and Stanford, 2008). Among all forms of cross-modal interactions, audiovisual processing remains a main vehicle for perceiving the events in the world (Koelewijn et al., 2010). Owing to the inherent higher temporal precision in auditory modality (Morein-Zamir et al., 2003; Alais and Burr, 2004), auditory events usually help to make the (noisy) visual stimuli perceptually distinctive and even pop-out among the cluttered environment. The influence of auditory signals upon visual events has been documented in a typical multisensory illusion – *temporal ventriloquism* (Shimojo et al., 2001; Bertelson and Aschersleben, 2003; Morein-Zamir et al., 2003; Vroomen and de Gelder, 2004; Burr et al., 2009; Cook and Van Valkenburg, 2009), which has been initially characterized by cross-modal capture of the perceived timing of visual events at the presence of concurrent auditory events.

Recently, demonstrations of temporal ventriloquism have been extended to dynamic contexts where different perceptual groupings compete to determine the final percept of visual apparent motion (Getzmann, 2007; Bruns and Getzmann, 2008; Freeman and Driver, 2008; Shi et al., 2010). For example, in the typical bouncing illusion, two balls moved across each other and elicited either streaming or bouncing off percept. Presentation of a ‘collision’ sound near the crossover of the balls facilitated the perception of ‘bouncing rather’ than ‘streaming’ (Sekuler et al., 1997; Shimojo et al., 2001). A few studies have used the combinations of a single auditory event with multiple visual events to investigate whether and how this single auditory event could selectively bind with only one of the multiple visual events; or alternatively, interacts with all the visual events, to reach a perceptual decision (simultaneity judgment or feature discrimination) on visual events (Van der Burg et al., 2008, 2013; Roseboom et al., 2009, 2013). The evidence so far suggests a ‘selective’ temporal ventriloquism effect, in which a subjective mapping from a single auditory event to multiple visual signals is hard to achieve.

The auditory stimuli used in previous studies were either a sequence of auditory beeps or a continuous sound lasting through the visual motion process, with the sounds being grouped in pitch or rhythm (Watanabe and Shimojo, 2001; Keetels et al., 2007; Bruns and Getzmann, 2008), obeying Gestalt principles such as proximity in frequency or time, continuous or smooth transition, onset and offset, rhythm and common spatial location (Bregman, 1990; Bregman et al., 1994; Carlyon, 2004). Nevertheless, the acoustic environment we live in usually consists of complex sound scenes. Multiple auditory inputs, if they are conflicted in features, would give rise to perceptual competition for human observers. Therefore, it is important to examine how we resolve the competition of perceptual organizations in the cross-modal interaction. Specifically, it is of ecological sense to examine whether and how the perceptual competition in a single modality (such as auditory modality) is resolved first to influence the visual percept; alternatively, it is important to examine how competitive sound signals directly (and selectively) interact with their visual counterparts by cross-modal grouping to obtain the final visual perceptual decision (Koelewijn et al., 2010; Talsma et al., 2010).

Here, we combined the auditory gap transfer illusion with the visual Ternus apparent motion to examine how perceptually competitive tones interact with the visual frames in resolving the ambiguous perceptual states of visual apparent motion. For the auditory stimuli, the gap transfer illusion refers to the auditory stimulus pattern that embodies a short descending (or rising) glide crossing with a long rising (or descending) glide. There is a short gap (around 100 ms) in the temporal middle of the cross in the long glide, the gap is yet perceived in the short gap instead of the long glide, by the perceptual integration of onsets and offsets of the sound segments at the crossing (Nakajima et al., 2000; Kanafuka et al., 2007). For the visual stimuli, we used a typical Ternus display that is composed of two successive visual frames, each containing two horizontal dots. The two frames share one common dot location when overlaid, with the other dot on the opposite side. With different inter-frame intervals (IFIs), participants can perceive two different percepts of apparent motion. When the IFI is short, ‘element motion’ (EM) is perceived with the outer dot moving from one side to the other, while the center dot remains static or flashing. However, long IFIs give rise to the perception of ‘group motion’ (GM), in which the two dots move together as a group (Ternus, 1926; Kramer and Yantis, 1997; Alais and Lorenceau, 2002; Petersik and Rice, 2008; Chen et al., 2010; Shi et al., 2010). Therefore, the perceived motion state of Ternus display is a function of the implicitly perceived IFI between two visual frames. As illustrated above, the auditory gap transfer illusion is composed of both physically and perceptually equal stimuli configurations. The visual Ternus display contains a relatively wide temporal range in categorizing the two motion percepts. The juxtaposition of auditory glides and visual Ternus display provides a good paradigm to examine the role of temporal perceptual groupings of auditory events upon visual motion, with easy manipulation of the temporal relations between auditory-visual events. Specifically, we here asked two empirical questions: (1) whether intra-modal temporal groupings precede over cross-modal interaction in the complex audiovisual scene; (2) whether the final visual percept is driven by the subjectively perceived temporal interval between visual frames when they interface with the ‘gap’ in the auditory glides.

To fulfill the research interests, four experiments were conducted. In Experiment 1A, participants were asked to make a two-forced choice toward the classification of visual Ternus apparent motion (EM vs. GM). The auditory stimuli consisted of the gap transfer and no gap transfer patterns, as well as a single ascending and a single descending glide tone (with or without a gap), together with the visual-only condition (Ternus display) as a control. As we noted, the direction of the pitch change (rising or falling) mattered significantly in the perception of dynamic loudness change (Neuhoff, 1998) and the rising tone takes perceptual priority with salient biological meaning (indicating the onset of a looming object) over decreasing tone (Neuhoff et al., 1999, 2002; Hall and Moore, 2003). In Experiment 1B, we manipulated the direction of pitch change (decreasing) to exclude any potential effect of the directional information of the pitch contour. To further examine the temporal correspondence between the perceived auditory gap interval and the ultimate visual motion percept, in Experiment 2A we curtailed the experimental conditions (from Experiment 1A) by selecting the critical stimuli configurations: auditory glides with gap transfer illusion, auditory gap with short glides, auditory gap with long glides and visual-only conditions. After completing the visual Ternus motion discrimination session, participants were required to compare visual intervals in different stimuli configurations. Gap intervals (50–230 ms) between two visual Ternus frames were embedded in the above three auditory stimuli configurations, with the standard IFI (140 ms) of visual Ternus frames (without accompanying tones).

To examine the potential mechanism of temporal proximity between auditory and visual events in modulating the perception of visual apparent motion, and to overcome the physical constraints (for cross-modal interaction) of larger temporal deviation between audiovisual events in Experiments 1 and 2, we implemented Experiments 3 and 4. Experiment 3 was implemented to address whether the temporal proximity between the visual frames and sound beeps (a gap in long glides) played an important role in modulating the percept of visual Ternus apparent motion by introducing two types of Ternus display (short frame duration vs. long frame duration). Experiment 4 was implemented to show how synchronous paired audiovisual events influenced the perception of visual Ternus motion. The results from the four experiments indicated that in a complex audiovisual interaction scenario, auditory (intra-modal) temporal grouping took priority over the audiovisual (cross-modal) temporal organization. Furthermore, the final decision for the percept of visual motion was largely determined by the perceived visual gap intervals.

## Experiment 1

### Method

Experiment 1 was conducted by using between-subjects design. The ascending glides were the long glides in Experiment 1A and the short glides in Experiment 1B. The descending glides were mirror-reversed in two experiments (short glides in Experiment 1A and long glides in Experiment 1B).

#### Participants

Twenty-seven undergraduate and graduate students (four females, with an average age of 24.9) participated in Experiment 1A. Twenty-eight students (eight females, with an average age of 23.3) took part in Experiment 1B. All participants reported having normal hearing and normal or corrected-to-normal vision. The experiments were performed in compliance with all institutional guidelines set by the Academic Affairs Committee, Department of Psychology at Peking University. All participants provided written informed consent according to the Declaration of Helsinki.

#### Apparatus and Stimuli

Visual stimuli were presented on a 17-inch CRT monitor (Viewsonic), controlled by a normal PC (HP AMD Athlon 64 Dual-Core Processor) with a Radeon 1700 FSC graphics card. The vertical refresh rate was set to 100 Hz and the resolution was 1024 × 768 pixels. The auditory stimuli (65 dB) were generated by a sound card (RME Fireface UFX) and binaurally presented to the participants’ ears with the headphone (Sennheiser HD 600). The computer programs for controlling the experiments were developed with Matlab (Mathworks Inc.) and the Psychophysics Toolbox (Brainard, 1997; Pelli, 1997). The test cabin was semi-anechoic and dimly lit throughout the experiment. The viewing distance was fixed at 60 cm, maintained by using a chin-rest.

#### Visual Stimuli

**FIGURE 1:**
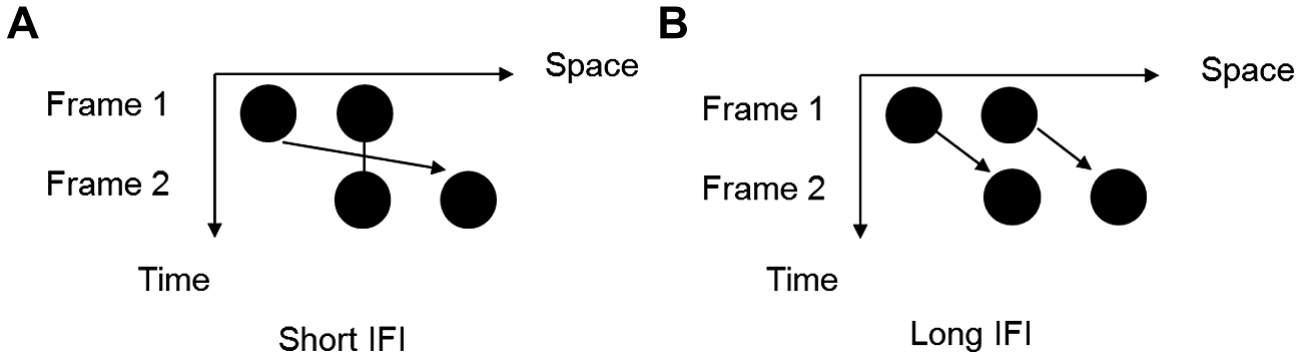
**Two possible motion perceptions of the Ternus display. (A)** ‘Element motion’: the center dot is perceived to remain at the same space, while the outer dot is perceived to move from one side to the other side; **(B)** ‘Group motion’: two dots are perceived to move together as a group.

**FIGURE 2:**
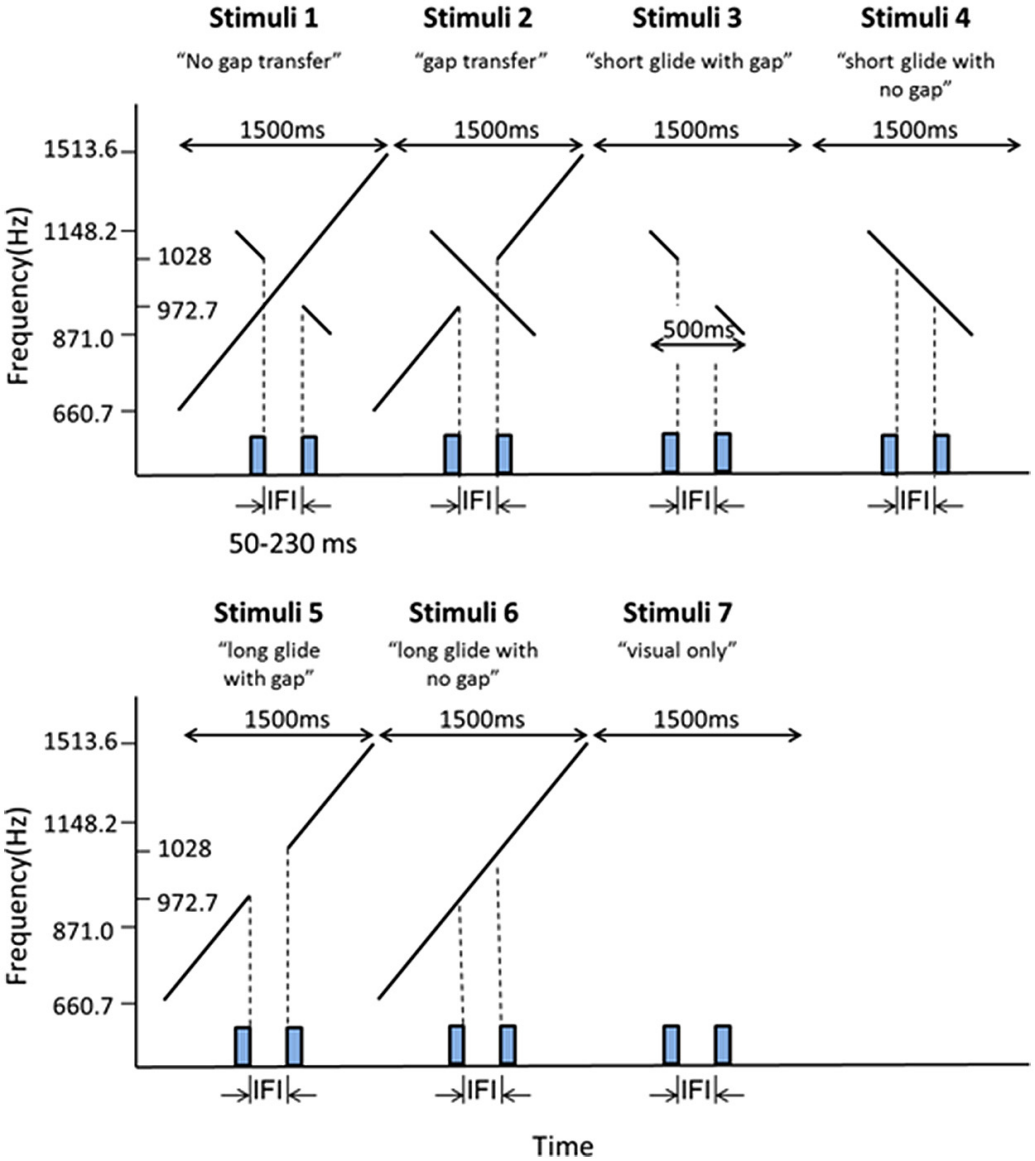
**Illustrations of the stimuli configurations for Experiment 1A.** Stimuli 1–6 consisted of both auditory and visual stimuli. Stimuli 7 contained only visual stimuli. The presentation duration of each stimulus configuration was 1500 ms. The rectangles on the X-axis in each stimuli pattern represented the two visual frames (30 ms for each frame) as shown in **Figure 1**. Stimuli 1 and 2 were composed of a short descending glide crossing over a long ascending glide. There was a gap in the temporal middle of the stimuli. The gap was in the short glide in the ‘no gap transfer’ stimulus configuration (Stimuli 1) and in the long glide in the ‘gap transfer’ stimulus configuration (Stimuli 2). The auditory stimuli in Stimuli 3–6 were the components of the stimuli configuration 1 and stimuli configuration 2, consisting of only the short glide or the long glide. Stimuli used in Experiment 1B were made by mirror-reversing the stimuli in Experiment 1A in time.

Visual stimuli were composed of two sequential stimulus frames, with each lasting 30 ms. Each frame contained two black horizontal dots presented on a gray background (10.6 cd/m^2^). The dots were 1.3° of visual angle in diameter, 0.24 cd/m^2^ in luminance and had a 2°of visual angle separation between them. The two frames shared one dot location at the center of the monitor with the two other dots located in the opposite positions relative to the center (**Figure 1**). The first frame ended at the beginning of the glide gap and the second frame started at the end of the gap (**Figure 2**), which meant the IFIs of the visual stimulus were equal to the gap intervals of the auditory stimulus.

#### Auditory Stimuli

Six auditory stimulus configurations and a control stimulus configuration (visual Ternus only) were used in Experiment 1A (**Figure 2**). In the first (A1-NGT: no gap transfer) and the second (A2-GT: gap transfer) stimulus pattern, the auditory stimuli consisted of a long ascending glide crossed with a short descending glide. The duration of the long ascending glide was 1500 ms, increasing from 660.7 to 1513.6 Hz, and the short glide lasted 500 ms, decreasing from 1148.2 to 871.0 Hz. Both glides moved linearly on the logarithmic frequency scale at a rate of 0.80 oct/s. The long and the short glide crossed each other at 1000 Hz with a temporally middle point at *t* = 750 ms from the beginning of the long ascending glide. At the crossing temporal point, “no gap transfer stimuli” (stimuli 1 configuration) had a temporal gap in the short descending glide, while the “gap transfer stimuli” had a gap in the long ascending glide. The rise and fall times were 150 ms at the beginning and end of the long glide, while the rise and fall times were 3 ms for the short glide (at the boundaries of the gap in either the long or the short glide).

In the third (A3-SG: short glide with gap) and fourth (A4-SNG: short glide with no gap) stimulus pattern, the auditory stimuli consisted of only the short glide with a gap (stimuli 3) or without a gap (stimuli 4). The short glide (A3, stimuli 3) was inserted with a gap, with the first auditory segment having frequencies of 1148.2–1028 Hz before the gap and the second segment of 972.7–871.0 Hz after the gap. The fourth stimulus pattern was a continuous glide with changing frequencies of 1148.2–871.0 Hz. The fifth stimulus pattern (A5- LG: long glide with gap) was a long glide with a gap, one auditory segment before the gap having frequencies of 660.7–972.2 Hz, with the other segment having frequencies of 1028–1513.6 Hz after the gap. The sixth stimulus pattern (A6-LNG: long glide without gap) consisted of the long glide without a gap, with pitch rising from 660.7 to 1513.6 Hz.

In Experiment 1B, the stimulus patterns were similar to those in Experiment 1A except that the auditory stimuli in the first six stimulus patterns were mirror-reversions of those in Experiment 1A. As a result, the directions of the pitch change for both the long and short glides were opposite to the configurations in Experiment 1A.

To accurately render the timing of the auditory and visual stimuli, the duration of the visual stimuli and the synchronization of the auditory and visual stimuli were controlled by the monitor’s vertical synchronization pulse.

#### Design and Procedures

Prior to the experiment, participants were shown demonstrations of ‘EM’ and ‘GM’ of visual Ternus display. They then practiced a series of trials. All participants reported clear discriminations between ‘EM’ and ‘GM’ with a correct response rate of about 95% after 60 trials.

A 7 (stimuli configurations: A1–A6, V-only) × 7 (IFI: 50, 80, 110, 140, 170, 200, or 230 ms) factorial design was adopted in Experiment 1A. Each auditory configuration had two blocks and each block had 42 trials. The directions of the apparent motion (left or right) were counterbalanced across the 42 trials. Totally, there were 14 blocks and 588 trials. Participants were required to fix their eyes on the center of the monitor and make discrimination of the perceptual state of visual Ternus display (‘EM’ vs. ‘GM’), ignorance of the auditory beeps if they were presented. A typical trial started with a fixation cross lasting for 300 ms. The stimuli appeared after a blank interval of 500 ms. In the auditory-present trials, participants heard the glides first and then saw the first visual frame, after a given ISI (from 50 to 230 ms), the second visual frame appeared. In the visual only trials, participants heard nothing but waited for the same time interval as that in the sound-present conditions before seeing the first visual frame. In the auditory stimuli present blocks, the IFIs of the visual frames were equal to those in the gap intervals. After all stimuli were presented, with a random delay of 300–500 ms, participants were presented with a question mark to make a two-forced choice response. They would press the left arrow for ‘GM’ and the right arrow for ‘EM.’

Experiment 1B was implemented with the same temporal structure and similar stimuli configurations as those in Experiment 1A, except that the time-frequencies of the glides were mirror-reversed accordingly.

In a control experiment, we presented participants with the auditory gap transfer stimuli pattern with different gap durations (stimuli 1 and 2), and asked 24 participants (10 females, aged from 23 to 30 years-old with an average age of 24.1 years-old) who attended in Experiment 1A to determine whether the short gap was in the long glide or the short glide. Nearly all participants perceived the short gap in the short glide rather than in the long glide. The averaged percentages for reporting the illusory gap in short glides with gap durations of 50 ms (i.e., gap50), gap80, gap110, gap140, gap170, gap200, and gap230 were 87.5 (with associated standard error of 3.7), 93.8 (2.4), 92.5 (2.6),93.3 (2.7), 93.8 (2.2), 90.0 (4.0), and 83.8 (5.7). Repeated measures analysis of variance (ANOVA; Greenhouse–Geisser corrected) showed a marginal significant difference for perceiving ‘illusory’ gap among the seven types of stimuli configurations, *F*(6,138) = 2.874, *p* = 0.063, with relatively lower percentages of reporting ‘gap transfer’ in the shortest gap (50 ms) and longest gap (230 ms) conditions. Those results suggested that the stimuli patterns used in the experiments deliver genuine gap transfer illusion properly.

### Results

**FIGURE 3:**
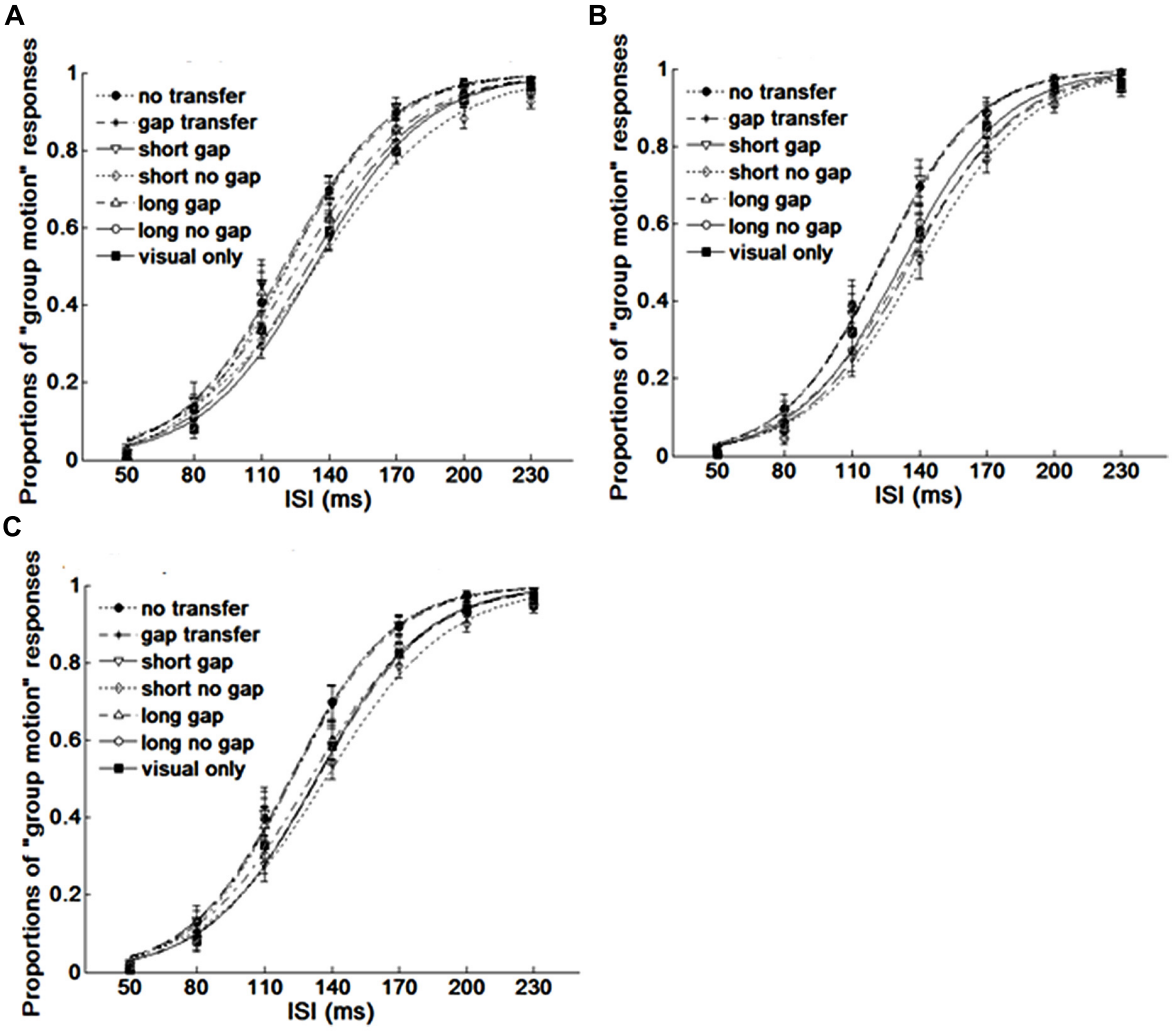
**Average psychometric curves for Experiment 1. (A) Experiment 1A, **(B)** Experiment 1B, **(C)** for averaged results of all participants.** Dot line with filled circles represents no gap transfer (A1-NGT); dot dash line with stars indicates gap transfer (A2-GT); Solid line with triangles short glide with gap (A3-SG); Dash line with diamonds short glide with no gap (A4-SNG); Dot dash line with triangles long glide with gap (A5- LG); Solid line with squares long glide with no gap(A6-LNG); Solid line with filled squares V-only condition. The error bars represent the associated standard errors.

**FIGURE 4:**
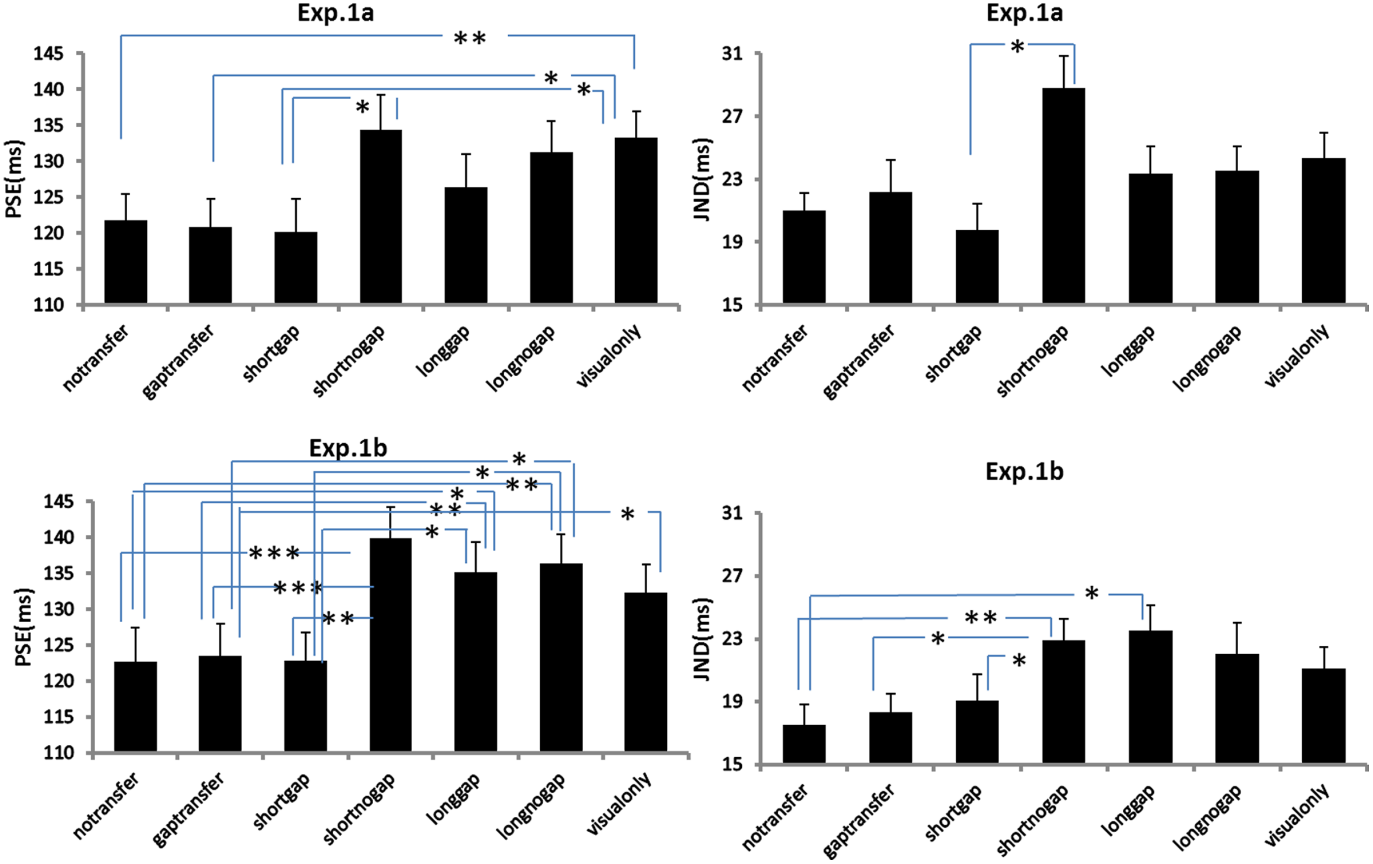
**Point of subjective equality (PSEs) and JNDs for Experiments 1A and 1B.** The error bars represent standard errors (^∗^*p* < 0.05; ^∗∗^*p* < 0.01; ^∗∗∗^*p* < 0.001).

The point of subjective equality (PSE) and just noticeable difference (JND) were calculated across each stimulus condition. PSE refers to the transitional temporal point at which percepts of ‘EM’ and ‘GM’ were perceived with equal probabilities, which can be calculated by estimating the point of 50% of the percentages for reporting ‘GM’ on fitted logistic function. JND represents the difference between the two motion perceptions, which is obtained by estimating the IFI difference between 50 and 75% of the GM responses from the psychometric curves (Treutwein and Strasburger, 1999). **Figure 3** shows the average psychometric curves in Experiments 1A,B for all participants. **Figure 4** shows the mean PSEs and JNDs (with associated standard errors) in Experiments 1A,B, which are also listed in **Table 1**.

**Table 1:** Point of subjective equality (PSEs) and JNDs and associated errors (ms) in Experiments 1A,B.

	Experiment 1A		Experiment 1B	
Auditory conditions	PSE (ms)	JND (ms)	PSE (ms)	JND (ms)
A1:No gap transfer	121.8 ± .3.6	21.0 ± 1.1	122.7 ± 4.7	17.5 ± 1.3
A2:Gap transfer	120.8 ± 3.9	22.1 ± 2.1	123.6 ± 4.5	18.3 ± 1.2
A3:Short no gap	120.1 ± 4.7	19.7 ± 1.7	122.9 ± 3.9	19.1 ± 1.6
A4:Short gap	134.3 ± 4.8	28.8 ± 2.1	139.9 ± 4.3	22.9 ± 1.4
A5:Long no gap	126.3 ± 4.7	23.3 ± 1.7	135.1 ± 4.2	23.5 ± 1.6
A6: Long gap	131.2 ± 4.4	23.5 ± 1.5	136.4 ± 4.0	22.0 ± 2.0
V-only	133.3 ± 3.6	24.3 ± 1.6	132.3 ± 3.9	21.1 ± 1.3

A repeated measures of ANOVA was conducted, with stimuli conditions (A1 ∼ A6 and V-only) as within-subjects factor and experiments (Experiments 1A or 1B) as between-subject factor. Both PSEs and JNDs were dependent factors. For PSEs, the main effect of auditory condition was significant, *F*(6,318) = 16.275, *p* < 0.001. However, no significant main effect of experiment was found, *F*(1,53) = 0.473, *p* = 0.495, neither the interaction between stimuli conditions and experiments, *F*(6,318) = 1.004, *p* = 0.423. Bonferroni correction (with 95% confidence interval for difference) was used for the full set of 21 possible pairwise comparisons. The analysis revealed no difference was found in PSEs between A1-‘no-gap transfer’ (122.3 ms), A2-‘gap-transfer’ (122.2 ms), and A3-‘short gap’ (121.5 ms), *ps* > 0.05, but they were smaller (*p*s < 0.05) than those in A4-‘short glide with no gap’ (137.1 ms), A5-‘long glide with gap’ (130.7 ms), A6-‘long glide with no gap’ (133.8 ms) and V-only (132.8 ms). PSEs in A4, A5, A6, and V-only were statistically equal, all *ps* > 0.1. Pairwise comparisons showed a notable effect of the short gap. The short glide with a temporal gap (physically- A3-SG or perceptually- A1-NGT and A2-GT) facilitated in separating visual frames and led to more reports of GM (with reduced PSEs).

On the other hand, ANOVA of JNDs also revealed a significant main effect of auditory conditions, *F*(6,318) = 6.322, *p* < 0.001. The main effect of experiments was not significant, *F*(1,53) = 2.711, *p* = 0.106. The interaction effect between auditory condition and experiment was also not significant, *F*(6,318) = 1.178, *p* = 0.318.

Bonferroni corrected comparison showed that JND in V-only (baseline, 22.7 ms) had no difference from those in A1 ∼ A6, all *ps* > 0.1. Nevertheless, there was a significant difference of JNDs among the auditory-present conditions (A1 ∼ A6), *F*(5,265) = 6.950, *p* < 0.001. JNDs were similar (*ps* > 0.05) in A1 (19.3 ms), A2 (20.3 ms) and A3 (19.4 ms), but were smaller (*ps* < 0.05) in A1 ∼ A3 than the JND in A4 (short no gap, 25.9 ms). Therefore, in general, participants’ sensitivities for discriminating the Ternus apparent motion remained nearly the same in both auditory present and absent conditions, while they showed noticeable lower sensitivities in the short glide with no gap.

## Experiment 2

Experiment 1 showed that short auditory glides with a gap (either physically or perceptually perceived) imposed a significant influence on the perception of Ternus motion (with a dominant percept of GM). However, long auditory glides with a gap had a less impact on the perceived ‘state’ of Ternus motion. Though, with the similar perceptual organization in both short glide and long glide, the modulation effects were different. It was probably due to the biased visual gap intervals (compared with the gap interval in the visual Ternus display) in different auditory configurations that further modulated the perceived states of visual apparent motion (Burr et al., 2009; Shi et al., 2010). In Experiment 2, we adopted three (characteristic) configurations of auditory glides from Experiment 1, i.e., gap transfer configuration (A2), short glide with a gap (A3) and long glide with a gap (A5). Compared with the standard interval (with fixed IFI = 140 ms as a gap in Ternus display). If the perceived gap interval in A5 is not biased in the presence of long glides, and meanwhile the perceived gap intervals in A 2 and A3 are longer and equivalent in terms of magnitudes of the illusory biases (Shi et al., 2010), we would observe the similar effect of auditory capture of visual Ternus motion – dominant ‘GM.’ However, the capture effect was less magnificent or even absent with the gap in long glides (A5), due to the less/null-bias in perceiving the gap interval in A5. Experiment 2 was hence implemented to test this hypothesis.

Participants first performed the classification task of Ternus apparent motion as they did in Experiment 1, they then did a time interval judgment task for discriminating the empty duration (gap) between two visual frames, in the presence of short/long glides (A2, A3, and A5), comparing the gap in between two visual Ternus frames (without accompanying short/long glides).

### Method

#### Participants

Eighteen undergraduate and graduate students (nine females, aged between 22 and 27; with an average age of 23.7) participated in Experiment 2. All participants reported having normal hearing and normal or corrected-to-normal vision and were naïve to the purpose of the study. The experiments were performed in compliance with all institutional guidelines set by the Academic Affairs Committee, Department of Psychology at Peking University. All participants provided written informed consent according to institutional guidelines and the Declaration of Helsinki.

#### Stimuli and Procedure

The experimental setting remained the same as that in Experiment 1, except that the stimuli conditions were reduced to four critical ones: A2 – GT (gap transfer), A3 – SG (short glide with a gap), A5 – LG (long glide with a gap), together with V-only (Visual only) condition. Participants performed two tasks: visual Ternus discrimination and gap interval judgment. In the visual Ternus discrimination task, the Ternus display was either presented together with the above three auditory configurations (A2, A3, and A5) or presented alone, the gap duration as well as the IFI between two visual frames was chosen from 50, 80, 110, 140, 170, 200, and 230 ms on a trial-by-trial basis. In the gap interval judgment task, the comparison stimuli were the gap intervals (50–230 ms) in Ternus display along with auditory configurations of A2, A3, or A5, while the standard stimulus was gap interval (with fixed IFI of 140 ms) in the Ternus display, in absence of any auditory sounds.

Prior to both tasks, participants received practice. Practice for the first task was the same with that in Experiment 1A and all participants reported clear discriminations between EM and GM. All the participants reported clear understanding of the tasks and the accuracy rates reached above 95% in both exercises for practice.

For the visual Ternus discrimination, participants essentially performed the same procedure as that in Experiment 1A except for a reduced number of auditory conditions as well as the number of trials. A block design with two factors was adopted: a 4 (stimuli condition: A2, A3, A5, V-only) × 7 (IFI: 50, 80, 110, 140, 170, 200, or 230 ms). The experimental procedures were the same with those in Experiment 1A.

After the visual Ternus discrimination task, participants continued to perform the task for gap interval comparison, although they were not informed of the experimental purpose in advance. For the visual gap interval judgment task, each auditory condition was presented for two blocks and each block had 56 trials, with a total number of 336 trials. The directions of the apparent motion (leftward or rightward) within one trial as well as the orders of the Ternus display with sound conditions and the Ternus display without sounds were counterbalanced. A typical trial started with a fixation point lasting for 300 ms. After a random delay of 500 ∼ 700 ms, the first visual Ternus frame was presented. After an inter-trial-interval (ITI) of 1 ∼ 1.2 s, the second visual Ternus frame was given. Then, after a blank waiting time of 300 ∼ 500 ms, participants were presented with a question mark to prompt them to make responses. They were asked to press the left- arrow key to indicate that they perceived the first display to have longer gap visual interval while the right arrow key for the opposite (the first having shorter interval). Participants were explicitly instructed to focus their attention on visual temporal interval, ignorant of the state of the visual apparent motion and the contextual sounds.

### Results

**FIGURE 5:**
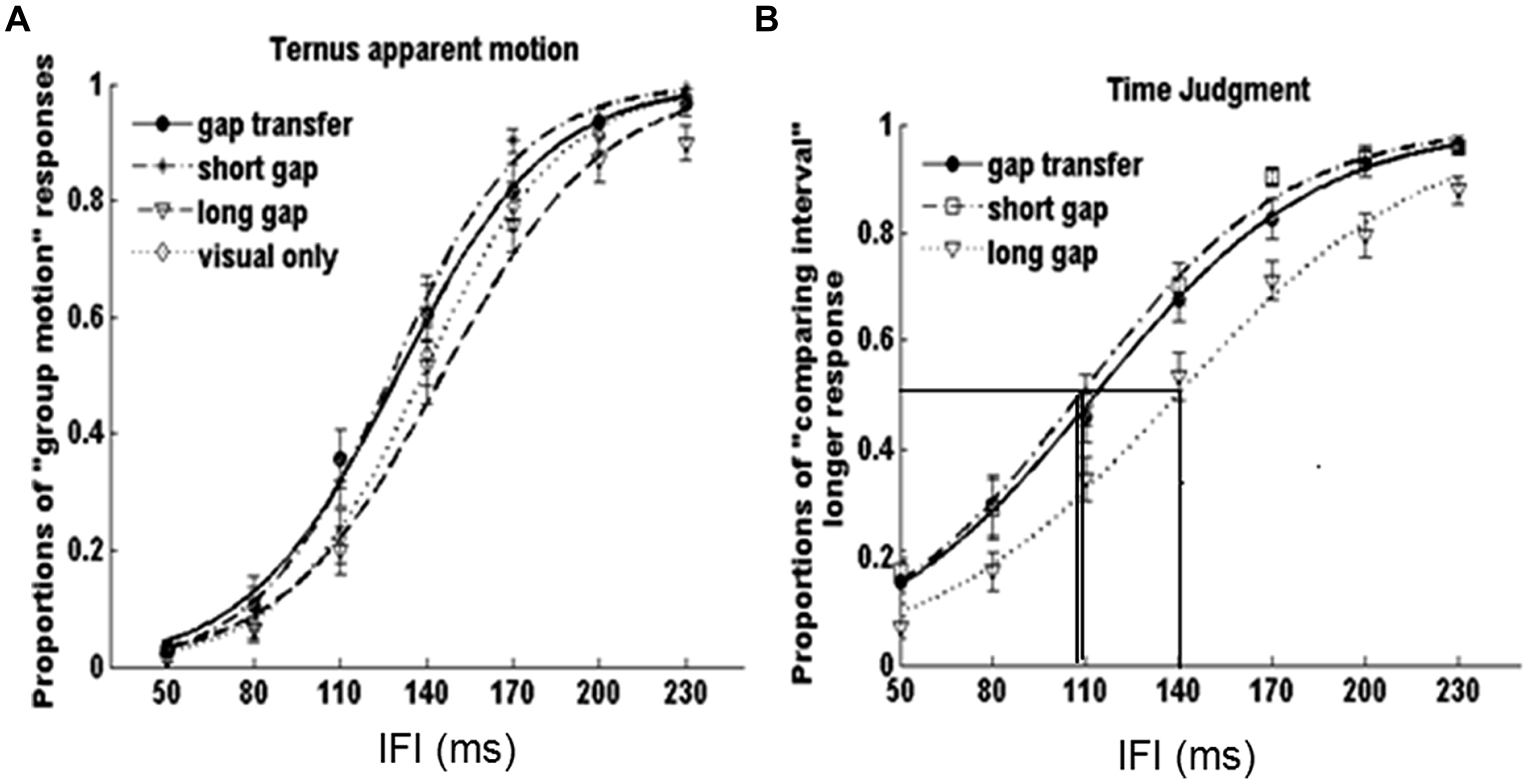
**Average psychometric curves in **(A)** Ternus apparent motion classification task and **(B)** time interval judgment task for all participants.** The error bars represent the associated standard errors, the three vertical lines in **(B)** indicated the PSEs for perceived gap durations in ‘gap transfer,’ ’short gap,’ and ‘long gap’ conditions.

**FIGURE 6:**
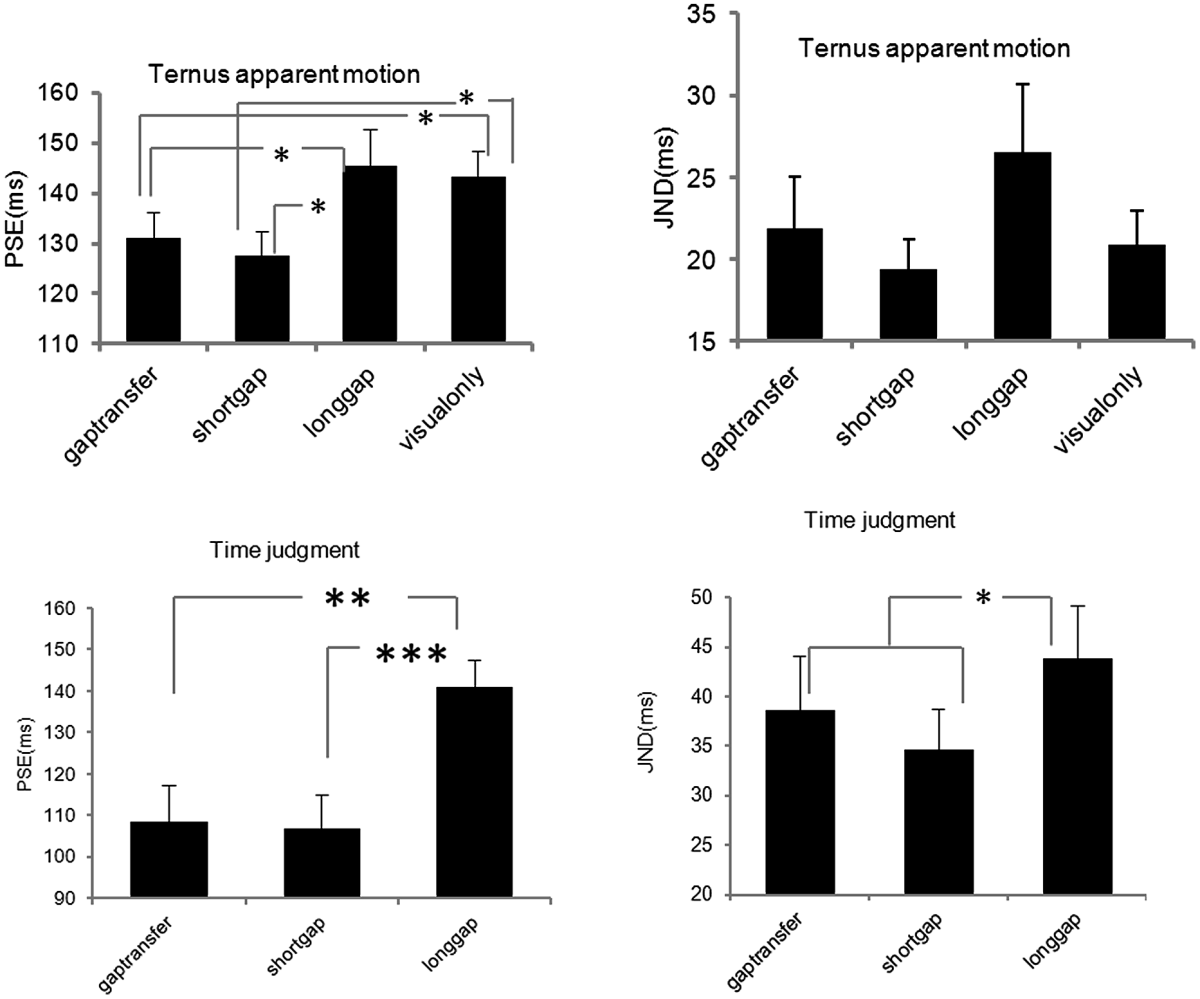
**Point of subjective equality and JNDs for Ternus apparent motion classification task and time interval judgment task in Experiment 2.** The error bars represent standard errors (^∗^*p* < 0.05; ^∗∗^*p* < 0.01; ^∗∗∗^*p* < 0.001).

**Figure 5** shows the average psychometric curves for both tasks. The values of PSEs and JNDs were also calculated (**Figure 5**). For the Ternus apparent motion task, the PSE values were as follows: 129.9 ± 4.5 ms for A2 – GT, 127.2 ± 4.7 ms for A3 – SG, 146.5 ± 5.9 ms for A5- LG, and 138.7 ± 4.4 ms for V-only. JNDs were 27.1 ± 4.0 ms for A2 – GT, 21.9 ± 1.8 ms for A3 – SG, 26.2 ± 3.1 ms for A5- LG, and 23.9 ± 2.3 ms for V-only. For the time interval judgment task, the PSE values were 109.7 ± 7.3 ms, 106.4 ± 6.5 ms, and 140.0 ± 5.6 ms, for A2, A3, and A5 respectively, and the JND values were 38.6 ± 4.7 ms, 34.7 ± 3.2 ms, and 43.8 ± 4.5 ms, for A2, A3, and A5 (**Figure 6**).

A repeated-measures ANOVA was conducted for both tasks. In the Ternus apparent motion classification task, ANOVA of PSEs and JNDs revealed the main effect of auditory conditions was significant, *F*(3,51) = 5.918, *p* < 0.05. Bonferroni corrected comparison confirmed the PSEs of A2-GT (146.5 ms) and of A3-SG (127.2 ms) were smaller (*ps* < 0.05) than those in A5- LG (146.5 ms) and V-only (138.7 ms) conditions, i.e., visual Ternus display accompanied with auditory gap interval (either in short auditory glide or in the auditory configuration with gap transfer illusion) led to a dominant percept of GM. However, there was no difference between A2 – GT (129.9 ms) and A3 – SG (127.2 ms), *ps* > 0.05, which suggested that the perceived auditory gap interval in the gap-transfer stimuli configuration exerted the same influence on the perception of visual apparent motion, as that did in the short glide with gap. ANOVA of JNDs revealed there was no significant main effect of auditory conditions, ruling out potential biased sensitivities of judging visual apparent motion when less conditions of auditory configurations were involved. The statistical results were consensus with the main findings in Experiment 1.

In the time interval judgment task, ANOVA of PSEs revealed the significant main effect of auditory condition, *F*(2,34) = 22.848, *p* < 0.001. Bonferroni corrected comparison confirmed the PSEs was the same with A2 – gap transfer (109.7 ms) and A3 – short glide with a gap (106.4 ms; *ps* = > 0.05), which were smaller than A5 – long glide with a temporal gap (140.0 ms; *p*s < 0.001), i.e., the ‘gap intervals’ between two visual Ternus frames in both gap transfer configuration and in short glide were subjectively perceived to last longer than the same physical visual interval in a long auditory glide. ANOVA of JNDs also revealed a significant main effect of auditory condition, *F*(2,34) = 4.608, *p* < 0.05. Bonferroni corrected comparison showed the JNDs was the same with A2 (38.6 ms) and A3 (34.6 ms; *ps* > 0.05), which were smaller than the JND in A5 (43.8 ms; *ps* < 0.05). The results suggested the gap interval in a long auditory glide was more difficult to discriminate than the gaps in short glides. Taken together, the findings confirmed the hypothesis proposed before: A2 (illusory perceived gap in short glides) and A3 (genuine physical gap in short glides) engendered the similar bias in perceiving gap interval and triggered the similar auditory temporal capture effect upon visual Ternus apparent motion.

## Experiment 3

In both Experiments 1 and 2, the weak effect of long glide tones (with a short gap in the middle) could be due to less temporal proximity between the onset of the first glide and the onset of first visual frame, as well as the larger deviance from the onset of the second visual frame to the offset of the second glide, although the offset of the first glide and onset of the second glide coincided with the onsets of the two visual frames respectively. The larger temporal deviance between ‘long’ glide and ‘short’ visual frames might constrain the strength of audiovisual perceptual grouping. Alternatively, the maintenance of the attention on the long glide could deteriorate the detection of ‘gap’ that makes the effect of gap upon the (blank interval between two) visual frames less significant. To examine above possibilities, as well as to address the concerns raised by the reviewers, we implemented Experiment 3, in which we manipulated the duration of the visual frame (short- 30 ms vs. long- 100 ms). Theoretically, we could select the duration of visual frame to be equivalent with the duration of segments of short glide (about 180 ms), or long glide (about 680 ms). However, as we learned from the pilot experiments and also from the literature (Kramer and Yantis, 1997; He and Ooi, 1999), Ternus frames with durations of 200 ms would trigger a potent percept of GM (a ceiling effect) even with a short IFI, which makes the modulation of glide less viable. Therefore, we chose a long duration (100 ms) of the visual frames, which was in temporal proximity to both onsets for short glides and long glides, compared with the short visual frames (30 ms in duration).

### Method

#### Participants

Sixteen students (nine females, aged between 16 and 25, with an average age of 22.2) participated in Experiment 3. All participants reported having normal hearing and normal or corrected-to-normal vision and were naïve to the purpose of the study. The experiments were performed in compliance with all institutional guidelines set by the Academic Affairs Committee, Department of Psychology at Peking University. All participants provided written informed consent according to institutional guidelines and the Declaration of Helsinki.

#### Stimuli and Procedure

**FIGURE 7:**
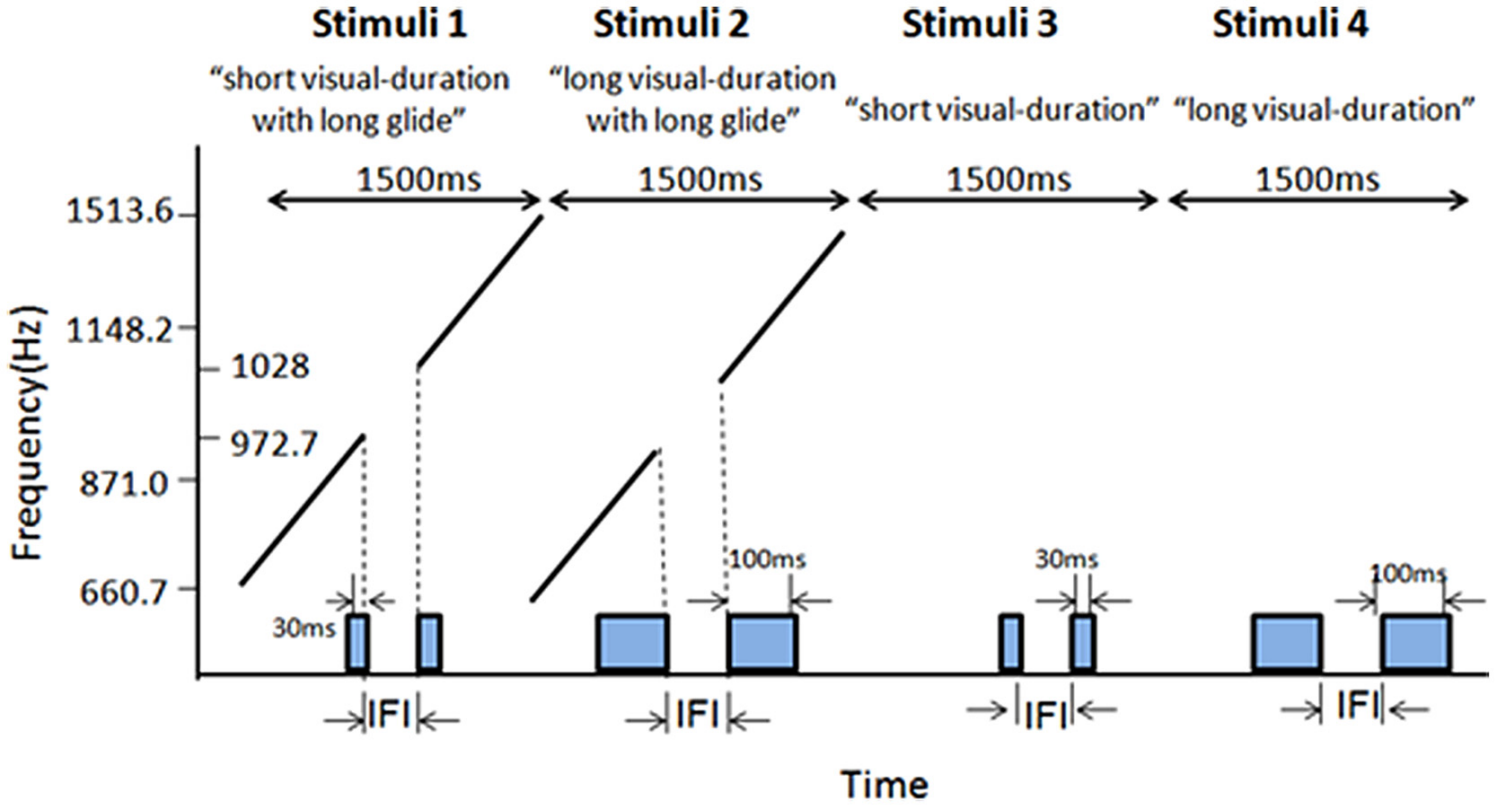
**Illustrations of the stimuli configurations for Experiment 3.** Stimuli 1 (‘short visual-duration with long glide’) and Stimuli 2 (‘long visual-duration with long glide’) consisted of a long auditory glide with a gap and a pair of visual frames. The duration of the visual Ternus frame was short in Stimuli 1, which was 30 ms, while in Stimuli 2 the visual Terns frame had a long duration of 100 ms. Stimuli 3 and 4 contained only visual frames. Stimuli 3 had a short visual duration (30 ms) and Stimuli 4 had a long duration (100 ms).

Experiment 3 adopted a 2 (Long glides: present or absent) × 2 (Visual durations: short or long) × 7 (IFI: 50–230 ms between Ternus frames) factorial design. In the long glide present condition, there was a long glide with a gap (50–230 ms), one auditory segment before the gap having frequencies of 660.7–972.2 Hz, the other segment after the gap having frequencies of 1028–1513.6 Hz after the gap (**Figure 2**, stimuli 5). In the long glide-absent condition, only visual Ternus stimuli were given. With the short visual duration, the visual Ternus frame was 30 ms as in previous two experiments. For the long visual duration, the duration of visual Ternus frame was 100 ms (**Figure 7**). The IFI between two visual frames was from 50 to 230 ms, with 30 ms as a step size.

Prior to the task, participants received practice. Practice was the same as that in Experiment 1 and all participants reported clear discriminations between EM and GM. All the participants reported clear understanding of the task and the practice reached an accuracy rate above 95%. For the formal test of visual Ternus discrimination, participants essentially performed the same procedure as in Experiment 1, with 560 trials.

### Results

**FIGURE 8:**
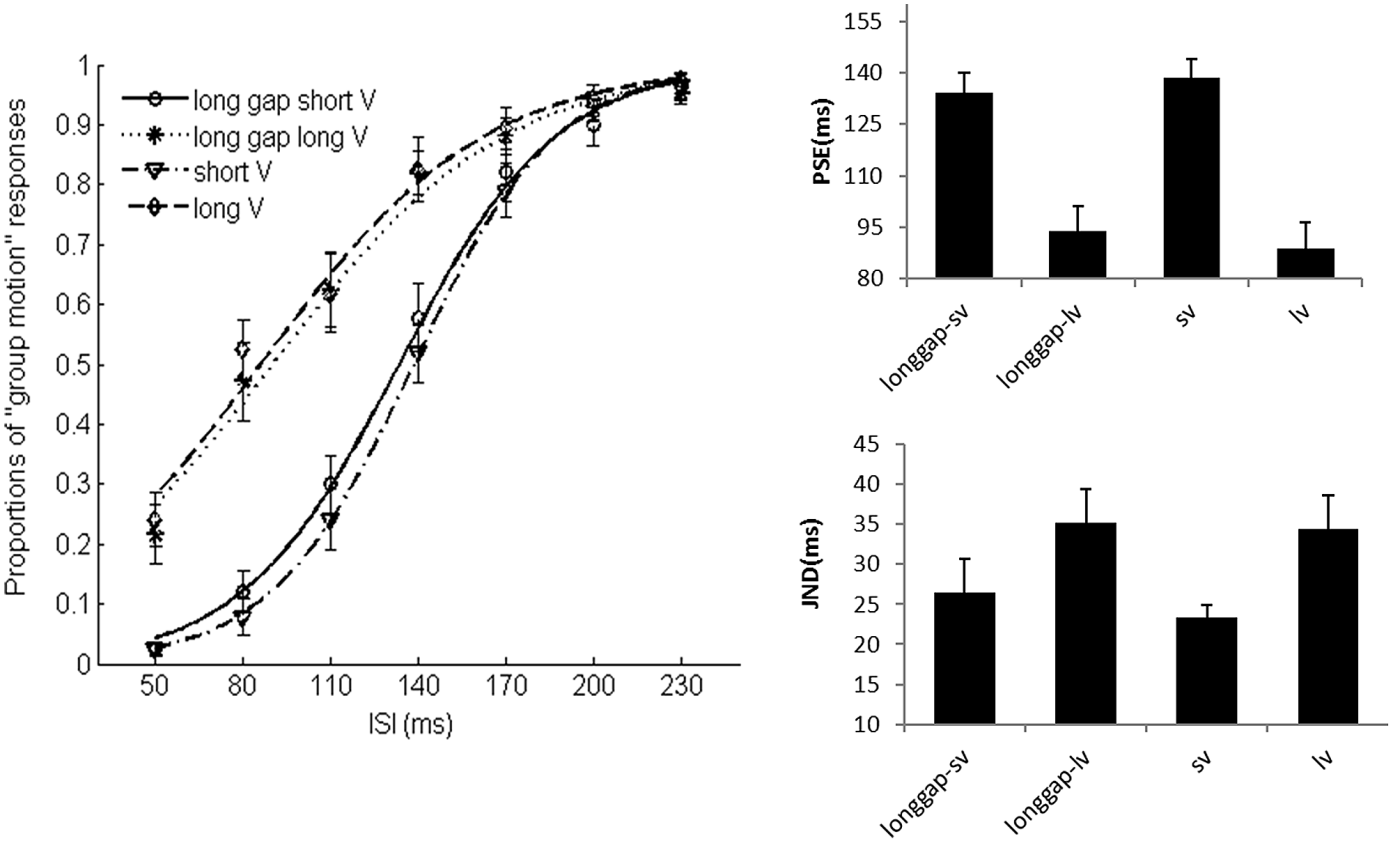
**Average psychometric curves, PSEs and JNDs for Ternus apparent motion classification task in Experiment 3.** The error bars represent the associated standard errors. *Long gap short V* (i.e., longgap-sv)- long glide with a gap and short visual frame; *Long gap long V* (longgap-lv)- long glide with a gap and long visual frame; *short V* (sv)- visual Ternus only condition, with frames of 30 ms; *long V*(lv)-visual Ternus only condition, with frames of 100 ms.

**Figure 8** shows the average psychometric curves, PSEs and JNDs for Experiment 3. The PSE values were: 138.5 ± 5.6 ms for short visual frame, 88.7 ± 7.9 ms for long visual frame, 134.1 ± 5.8 ms for long glide-gap with short visual frame, 93.8 ± 7.5 ms for long glide-gap with long visual frame. A 2 (visual duration: short or long) × 2 (long glide: present or absent) repeated measures ANOVA showed a main significant effect of the visual duration, the mean PSE for short visual frame was 136.3 (5.1) and 91.2 (6.8) for long visual frame, *F*(1,15) = 92.717, *p* < 0.001. However, the main effect of auditory glide- present or not was not significant, *F*(1,15) < 1, mean PSEs were 114.0 (5.8), 113.6 (6.3) for glide-present and glide-absent conditions. There was no interaction effect between visual duration and glide presence, *F*(1,15) = 1.551, *p* > 0.05.

The JNDs were: 23.3 ± 1.6 ms for short frame, 34.4 ± 4.2 ms for long frame, 26.5 ± 4.2 ms for long glide-gap with short visual frame and 35.2 ± 4.2 ms for long glide-gap with long visual frame. A 2 (visual type: short or long) × 2 (long glide: present or absent) repeated measures ANOVA showed a main significant effect of the visual duration, the mean JND for short visual frame was 24.9 (2.4) and 34.8 (4.0) for long visual frame, *F*(1,15) = 17.423, *p* < 0.01. However, the main effect of the factor with glide present or not was not significant, *F*(1,15) < 1; the mean JNDs were 30.9 (4.0), 28.8 (2.6) for glide present and glide absent conditions. There was no interaction effect between the factors of visual duration and glide, *F*(1,15) = 0.614, *p* > 0.05.

Taken together, long duration of visual frames led to dominant perception of GM (with lower PSEs) but decreased the sensitivity of discriminating Ternus motion (with higher JNDs). However, the manipulation of the long glide with a gap did not modulate the percept of visual Ternus motion.

In above Experiments, we fixed the time parameters for auditory glides. To examine the role of temporal proximity between audiovisual events in modulating the perception of visual Ternus motion, indeed there was a physical (duration) limitation of the visual stimuli. As we have seen in Experiment 3, the longer duration of the visual frames would lead to a ceiling effect for reporting ‘GM,’ and we technically shortened the duration to 100 ms to show how potentially the (long) auditory glides would modulate the (long) visual frames. However, one may notice that the temporal relation between auditory and visual events was reciprocal. In Experiment 4, we fixed the durations (30 ms) of the visual Ternus frames but manipulated the durations of the paired sound beeps (20 vs. 60 ms, with fixed pitch). In difference to the larger temporal deviance between the onsets of visual events and auditory glides in Experiments 1–3, the onsets of auditory beeps (sound markers) and visual frames were synchronized. Therefore, we can further examine the role of temporal proximity in modulating the perception of visual apparent motion.

## Experiment 4

In Experiment 4, we included three conditions: visual Ternus only (with frame duration of 30 ms, IFIs of 50–230 ms), visual frames with synchronized short paired beeps (1000 Hz, duration of 20 ms) of proximate duration as the visual frame, and visual frames with synchronized long paired beeps (1000 Hz, duration of 60 ms) of less proximate duration as the visual frame. If the temporal proximity between visual duration and auditory duration is critical in influencing the percept of visual apparent motion, we would expect a larger modulation effect of sound markers with 20 ms (i.e., more approximate to 30 ms- visual duration) than do sound markers with 60 ms duration (i.e., less approximate to 30 ms of visual duration). We also implemented an interval discrimination task, in which participants were required to make comparison of the perceived empty interval between two visual Ternus frames in the present of paired short beeps (20 ms) and paired long beeps (60 ms), to show whether the perceived time intervals contribute to the potential different percept of visual apparent motion.

### Method

#### Participants

Eleven students (nine females, aged between 22 and 30 with an average age of 26.6) participated in Experiment 4A (Discrimination of visual apparent motion). Ten of them attended Experiment 4B (Comparison of visual interval in the presence of sound beeps). All the screening procedure for the participants and requirement of the informed consent were the same as in Experiment 3.

#### Stimuli and Procedure

**FIGURE 9:**
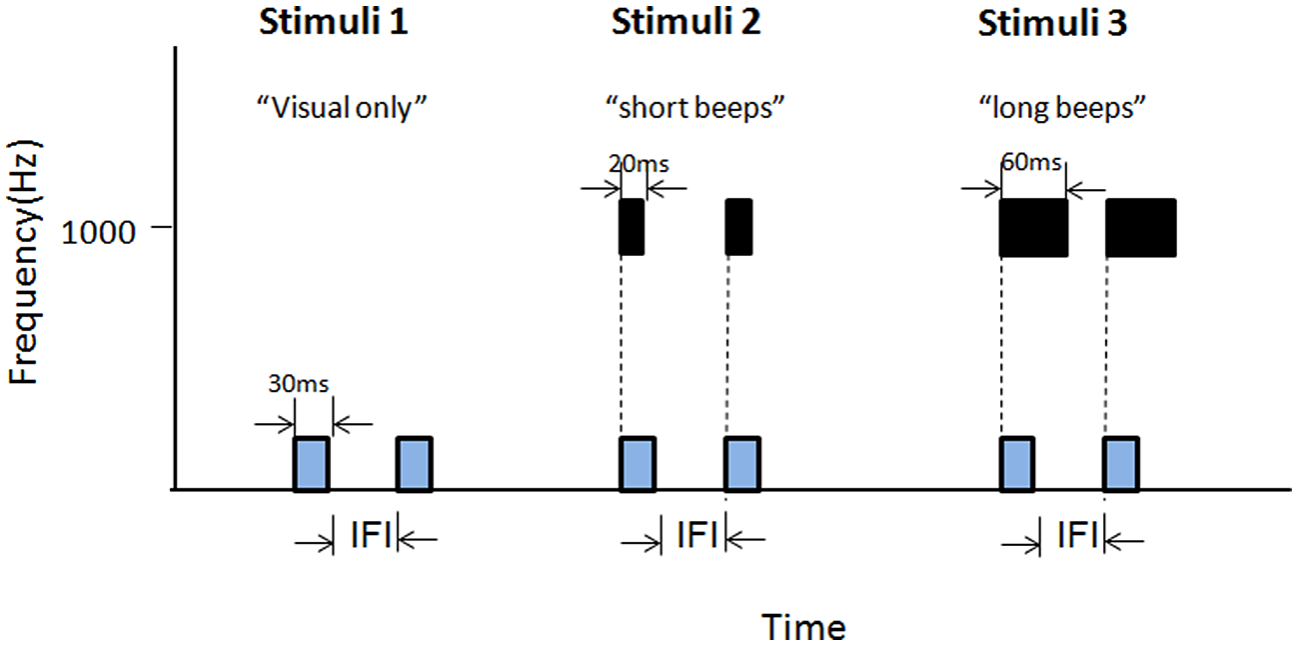
**Illustrations of the stimuli adopted in Experiment 4A.** Stimuli 1 contained only visual Ternus frames. Stimuli 2 consisted of a pair of short auditory beeps (1000 Hz, 30 ms) synchronous with the visual frames. Stimuli 3 composed of a pair of long auditory beeps (60 ms) synchronous with the visual frames.

Experiment 4A introduced two types of paired beeps. We adopted 3 (stimuli types) × (IFIs) factorial design. The stimuli types included visual-only, visual Ternus frames synchronous with paired short beeps (20 ms in duration), and visual Ternus frames synchronous with paired long beeps (60 ms in duration; see **Figure 9**). The IFI between two visual frames was from 50 to 230 ms, with 30 ms as a step.

Prior to both tasks, participants received practice as in Experiment 3. For the formal test of visual Ternus discrimination, participants essentially performed the same procedure as they did in Experiment 3, ignorant of the presence of the sound beeps. They then attended the formal test of Ternus motion discrimination, with total trials of 432. Afterward, they performed the comparison of the intervals between visual Ternus frames, enclosed by a pair of short tones (20 ms, standard interval: 160 ms) or a pair of longer tones (60 ms, comparison interval: 70–250 ms). The order of the standard stimuli and comparison stimuli were randomized and counterbalanced. The number of the trails was 336.

### Results

**FIGURE 10:**
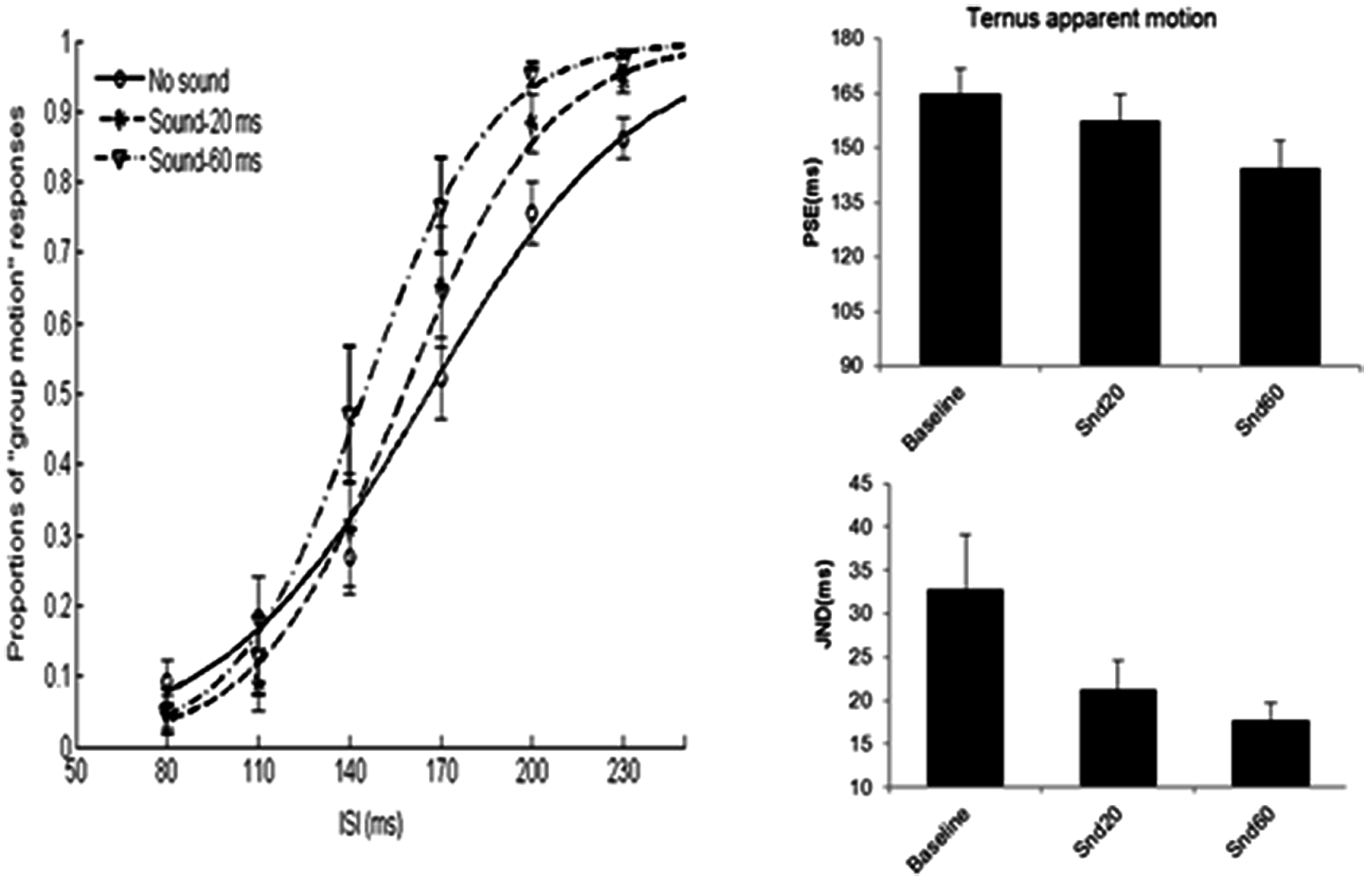
**Average psychometric curves, PSEs and JNDs for Ternus apparent motion classification task in Experiment 4.** *No sound*-baseline condition for visual Ternus motion. *Sound-20 ms* indicated visual Ternus frames were enclosed by a pair of short sound markers (20 ms in duration), *Sound-60 ms* indicated visual Ternus frames with a pair of long sound markers (60 ms in duration). The error bars represent the associated standard errors.

**Figure 10** shows the average psychometric curves, PSEs and JNDs for Experiment 4A. The PSE values were: 164.9 ± 6.8 ms for baseline (visual only baseline), 157.4 ± 7.3 ms for short sound marker (denoted as ‘Snd20’), 144.6 ± 7.4 ms for long sound marker (‘Snd60’). Repeated measures of ANOVA with PSEs showed a main significant effect of the stimulus type, *F*(2,20) = 14.862, *p* < 0.001. Bonferroni-corrected comparison showed significant differences between baseline and snd60, between snd20 and snd60, *p*s < 0.01. However, there was no difference between baseline and snd20, *p* = 0.478.

The JND values were: 32.8 ± 6.3 ms for baseline (visual only baseline), 21.2 ± 3.4 ms for short sound marker (‘Snd20’), and 17.7 ± 2 ms for long sound marker (‘Snd60’). Repeated measures of ANOVA showed a main significant effect of the stimulus type, *F*(2,20) = 9.630, *p* < 0.01. Bonferroni-corrected comparison showed significant differences between baseline and snd20, *p* < 0.05, between baseline and snd60, *p* < 0.05. However, there was no difference between snd20 and snd60, *p* = 0.348.

One sample *t*-test with the standard interval of 160 ms, showed the duration of the empty interval enclosed by long sound marker was perceived to last longer than the one enclosed by short sound marker, PSE = 116.6 ± 6.5 ms, *t*(9) = -6.648, *p* < 0.001.

Taken together, the data showed that sound pair with a longer duration (60 ms) imposed a stronger modulation effect upon the visual Ternus motion than sound pair with a short duration (20 ms) did. Moreover, the empty interval enclosed by long marker was perceived to last longer than the one enclosed by short marker (Tse et al., 2004).

## General Discussion

In this study we examined how competitive auditory inputs modulated the ambiguous percept of visual apparent motion. We extended the investigation of the relation between auditory grouping and multisensory integration from simple auditory stimuli configurations to more complex and perceptually competitive auditory environment.

### Intra-Modal Grouping vs. Cross-Modal Grouping

In current stimulus configurations, the overlapping of ascending and descending continuous auditory glides gave rise to perceptually competing edges of the temporal gap (i.e., gap transfer illusion). When the visual Ternus display was embedded in the glides, our perceptual system had to resolve the correspondence between the visual frames and the multiple auditory segments. Perceptual grouping in terms of correspondence could take two modes: (1) Intra-modal (auditory) grouping precedes over cross-modal perceptual organization. In this mode, observers would perceive the gap ‘transfer’ from the long glide to the short glide, the readout of the illusory gap within the short glide then imposes an influence upon the perception of visual apparent motion. (2) Cross-modal (auditory-visual) grouping takes priority over intra-modal perceptual organization. By this operation, on the contrary, the components of the auditory glide that were temporally close to the visual frames of the Ternus display would play a major role in interacting with the visual stimuli and gave rise to the final percept of visual Ternus motion. Take the stimuli 2- ‘gap transfer’ configuration (**Figure 2**) for instance, if the intra-modal grouping takes in priority, the perceived illusory gap as a product of the intra-modal perceptual organization in the short glide, would ventriloquize (pull) the two visual frames, leading to a dominant percept of ‘GM.’ Otherwise, if the element auditory glide independently interacts with the visual frames, the short continuous glide would fill in the interval gap in Ternus frames, triggering a continuous visual motion (Getzmann, 2007; Bruns and Getzmann, 2008) – i.e., a dominant percept of ‘EM,’ rather than discontinuous motion- ‘GM’; On the other hand, the interval gap in the long auditory glide (or the two ‘broken’ auditory segments) would correspond to each visual frame, leading to a dominant percept of GM (Shi et al., 2010). Therefore, the two potentially opposite and competitive cross-modal organizations would interact to render a null (bistable), if any, weak percept of either ‘EM’ or ‘GM.’ However, the experimental results showed that a dominant percept of ‘GM’ was reported, largely supporting a priority of intra-modal (auditory) perceptual grouping process that pit against the cross-modal (auditory-visual) perceptual grouping.

### The Capture Effect is Mostly a Result of Gestalt Temporal Grouping Temporal Phenomenon

Interestingly, the auditory facilitation effect was found for the short glide with a gap condition (A3- SG) but was not observed in the long glide with a gap (A5- LG), although the event structures for both sound conditions were essentially the same. We accounted this differential finding as a temporal perceptual grouping phenomenon. The perceived visual gap interval in the Ternus display was modulated by different auditory stimuli configurations. The visual gap intervals within the long glides/beeps were perceived to last shorter than those enclosed by short glides/beeps (Experiments 2B and 4B), and the perceived visual gap intervals were congruent with the results of Ternus motion classifications (more reports of ‘GM’ in Experiments 2A and 4A). Therefore, auditory capture on visual apparent motion was mainly dependent on the perceived visual interval between Ternus frames. The present result was consensus to our former findings (Shi et al., 2010). One might argue that the temporal proximities between the visual frames and the segment auditory glides determined the bias for perceiving visual apparent motion. However, the temporal proximity played a limited role in this bias. As is shown in Experiment 3, even for visual Ternus frames with longer durations (100 ms), when they were in closer temporal proximity to the long glide segment than were visual Ternus frames of short durations (30 ms), the perceptual grouping between (long) glides and (relatively long) visual frames did not lead to a potent bias for judging the states of Ternus motion. In the same vein, visual Ternus frames (20 ms) enclosed by short auditory markers (30 ms) did not elicit more reports of ‘’s than did long auditory markers (60 ms), although the temporal disparity between audiovisual events was larger for the latter condition. Taken together the results from Experiments 1–4, it indicated that the strength of inter-sensory binding, rather than the single factor of temporal proximity between audiovisual events, contributed to resolving the ambiguity of visual apparent motion. Moreover, temporal illusion due to the mechanism of selective attention, also contributed to the modulation effect by the gap (or illusory gap) inside the short auditory glides. We discussed this point as follows.

### Potential Selective Attentional Mechanism

Cross-modal integration in the short glide configurations could take place pre-attentively by a stimulus-driven, bottom–up process. In the short glides, the auditory signals remained sparse and made the gap to be salient. The attentional selection of the auditory gap was automatic and facilitated the cross-modal audiovisual perceptual grouping, by a reversed effectiveness principle (Stein and Stanford, 2008). The salient gap could elicit a neural response that was strong enough to be automatically linked to/freeze with the weaker/ambiguous neural response to visual stimuli in Ternus display, and hence biased the percept of Ternus apparent motion.

However, under the circumstance when the single glide was long, the maintenance of attention on the long auditory markers was deteriorated along the unfolding of the glides. The first marker, has inhibited the detection/perception of the short gap (Penner, 1977). As a result, the embedded short gap did not receive enough attentional focus to pop out from the continuous single glide and thus attenuated the facilitation effect on visual motion perception. Findings from Experiment 2 supported this hypothesis. In Experiment 2, temporal interval judgment toward the gap within long glides showed a less bias for the perceived duration, compared with the perceived magnificently longer (illusory) gap within short glides. This result pattern indicated a larger attentional focus on short glides and a less attentional focus on the gap enclosed by long glides (Tse et al., 2004).

On the other hand, a process of top–down attention might engage in the cross-modal interaction with more complex audiovisual stimuli. In the gap transfer (A1) or no gap transfer (A2) stimuli configurations, with both ascending and descending glides, multiple stimuli (including auditory glides and visual stimuli) were competing for the attentional processing resources. The complex scenario required the limited attention resources to be deployed across the multiple stimuli, so that observers could determine which component stimulus was to be selectively processed. Results from both A1-NGT and A2-GT conditions showed that even in the complex auditory scene, our brain could adaptively deploy the attention and select the signature gap by ignoring the other auditory glides, showing a selective temporal ventriloquism effect (Roseboom et al., 2009, 2013).

Nevertheless, currently we are not informed of whether more complex auditory environment (say, with more auditory glides) would constrain the capacity of audiovisual integration (Van der Burg et al., 2013). Furthermore, since multisensory integration takes an optimal temporal window to develop (Stein and Stanford, 2008) and attention needs some time to engage/deploy (Woodman and Luck, 1999), for future study, we need deep investigations into the time course of how attention interplays with cross-modal integration to resolve the ambiguous percept of visual motion.

## Conflict of Interest Statement

The authors declare that the research was conducted in the absence of any commercial or financial relationships that could be construed as a potential conflict of interest.
